# Roadside Survey of Ants on Oahu, Hawaii

**DOI:** 10.3390/insects9010021

**Published:** 2018-02-11

**Authors:** Reina L. Tong, J. Kenneth Grace, Paul D. Krushelnycky, Helen Spafford

**Affiliations:** College of Tropical Agriculture and Human Resources, University of Hawaii at Mānoa, 3050 Maile Way, Gilmore Hall 310, Honolulu, HI 96822, USA; kennethg@hawaii.edu (J.K.G.); pauldk@hawaii.edu (P.D.K.); hspaffor@hawaii.edu (H.S.)

**Keywords:** Formicidae, ant distribution, convenience sampling

## Abstract

Hawaii is home to over 60 ant species, including five of the six most damaging invasive ants. Although there have been many surveys of ants in Hawaii, the last island-wide hand-collection survey of ants on Oahu was conducted in 1988–1994. In 2012, a timed hand-collection of ants was made at 44 sites in a systematic, roadside survey throughout Oahu. Ants were identified and species distribution in relation to elevation, precipitation and soil type was analyzed. To assess possible convenience sampling bias, 15 additional sites were sampled further from roads to compare with the samples near roads. Twenty-four species of ants were found and mapped; *Pheidole megacephala* (F.), *Ochetellus glaber* (Mayr), and *Technomyrmex difficilis* Forel were the most frequently encountered ants. For six ant species, a logistic regression was performed with elevation, average annual precipitation, and soil order as explanatory variables. *O. glaber* was found in areas with lower precipitation around Oahu. *Paratrechina longicornis* (Latrielle) and *Tetramorium simillimum* (Smith, F.) were found more often in lower elevations and in areas with the Mollisol soil order. Elevation, precipitation, and soil type were not significant sources of variation for *P. megacephala, Plagiolepis alluaudi* Emery, and *T. difficilis*. *P. megacephala* was associated with fewer mean numbers of ants where it occurred. Ant assemblages near and far from roads did not significantly differ. Many species of ants remain established on Oahu, and recent invaders are spreading throughout the island. Mapping ant distributions contributes to continued documentation and understanding of these pests.

## 1. Introduction

Ants (Hymenoptera: Formicidae) are complex social insects considered by Hölldobler and Wilson to be the “culmination of insect evolution” [1]. Ants thrive in a range of environments and are often ecologically important [1]. These diverse, social insects provide several ecosystem functions, such as soil turnover and nutrient redistribution, and serve as prey for a variety of predators including mammals, reptiles, spiders [2], and other ants [1]. Ants also act as predators, seed dispersers, scavengers, and detritivores [2,3]. As such influential and ubiquitous insects, ants affect agriculture, the economy, biodiversity, and human and vertebrate health [2,4,5,6,7,8,9]. Invasive ant species, in particular, have the potential to adversely affect areas to which they are introduced [2].

### Ants in Hawaii

Hawaii is the most isolated island archipelago in the world, 3200 km from any other island and 4000 km from the nearest continent [10]. Only half the insect orders and about 15% of insect families are represented in Hawaii’s native terrestrial fauna [10]. Wilson and Taylor [11] consider the ant fauna of Hawaii to be completely introduced. Multiple surveys of ants in Hawaii have been conducted and these have documented an increase in species over time, and ants are now found throughout the islands, with most limited to lower elevations [6,12]. To date, at least 60 species of ants have become established in Hawaii [13] including five of the six most damaging invasive ants, *Anoplolepis gracilipes* (Smith), long-legged ant; *Linepithema humile* (Mayr), Argentine ant; *Pheidole megacephala* (F.), big-headed ant; *Solenopsis geminata* (F.), tropical fire ant; and *Wasmannia auropunctata* (Roger), little fire ant [2,6]. Ten species of ants are considered to be of public health concern on Oahu: *Camponotus variegatus* (Smith), *L. humile*, *Monomorium pharaonis* (L.), *Monomorium floricola* (Jerdon), *Ochetellus glaber* (Mayr), *Paratrechina longicornis* (Latreille), *P. megacephala*, *Pseudomyrmex gracilis* (F.), *S. geminata* and *Tapinoma melanocephalum* (F.) [7,8].

Ant surveys in Hawaii have been conducted sporadically over time and tend to use a variety of methods, including identification of museum specimens, use of historical data, hand collection, pitfall traps and bait cards, and the area surveyed has also varied (e.g., from multiple, whole-island surveys to commercial fields). In 1899, Forel [14] reported twenty species of ants in Hawaii, three of which were later identified as synonymous with another species, mislabeled, or no longer occurring in Hawaii [15]. Gulick [16] prepared a synoptic list and key to 23 ants in Hawaii in 1913. Wheeler [15], through examination of museum specimens in addition to his own collection, presented a revised list of 35 ant species for Hawaii in 1934. Phillips [17] surveyed ants in pineapple fields. Wilson and Taylor’s “Ants of Polynesia” monograph from 1967 [11] lists 36 species of ants in Hawaii, 34 occurring on Oahu. Huddleston and Fluker [18] found 42 species of ants in Hawaii in an archipelago-wide hand-collection survey published in 1968. Reimer, Beardsley, and Jahn presented the distributions of 41 species of ants by island [19] in 1990 from historical data and collections by authors. In 1994, Reimer presented the distribution of 38 ants by ecological communities from historical data [12]. In 2005, Krushelnycky [6] reported 47 species of ants established in Hawaii based on a review of historical data. Leong and Grace [7] analyzed Hawaii Department of Health Vector Control Branch inspection reports from 1990 to 1999 to evaluate pest occurrences on Oahu, with the most ant complaints related to *M. pharaonis*, *C. variegatus*, and *O. glaber* [7]. Ant surveys in montane natural areas [20] and in high-risk ports of entry [e.g., airports, military bases, etc., [21]] continue to be conducted on Oahu. In one of the most recent intensive surveys of ants in Hawaii, Brigham Young University (BYU) faculty and students collected ants on Oahu from 1988 to 1994 using roadside hand-collection techniques [22], finding 32 species from 280 locations; this data was published in 2012. Currently, 41 species of ants are known to occur on Oahu [19]. 

The objective of this study is to provide an updated descriptive distribution of ants from roadsides on Oahu, investigate environmental factors that may influence their distribution, and to find possibly newly established ant species. Mapping ant distributions on Oahu may reveal species interactions, help inform possible management decisions, and contribute to the understanding of the factors that influence species distributions [6]. Information regarding alien ants may be useful to other geographic areas experiencing invasions [12]. Finally, the current survey will also provide reference data for future comparisons.

Possible bias from convenience sampling (drawing samples from part of the population close to hand) [23] was a concern during planning for our survey. Sampling site disturbance from proximity to roads may include pedestrian traffic, vegetation maintenance (e.g., mowing, landscaping, tree-trimming, etc.), and chemical inputs (e.g., fertilizers, herbicides, pollution from vehicles, etc.). Roads may also alter surrounding soil pH, organic material, and favorability to native vegetation [24]. Road edges are more likely to harbor exotic vegetation [24]. In addition, species richness might be affected due to disturbance [25,26]. To examine whether these factors may influence survey results, our sampling also included the collection of ants a short distance from the roadside for comparison.

## 2. Materials and Methods

**Figure 1:**
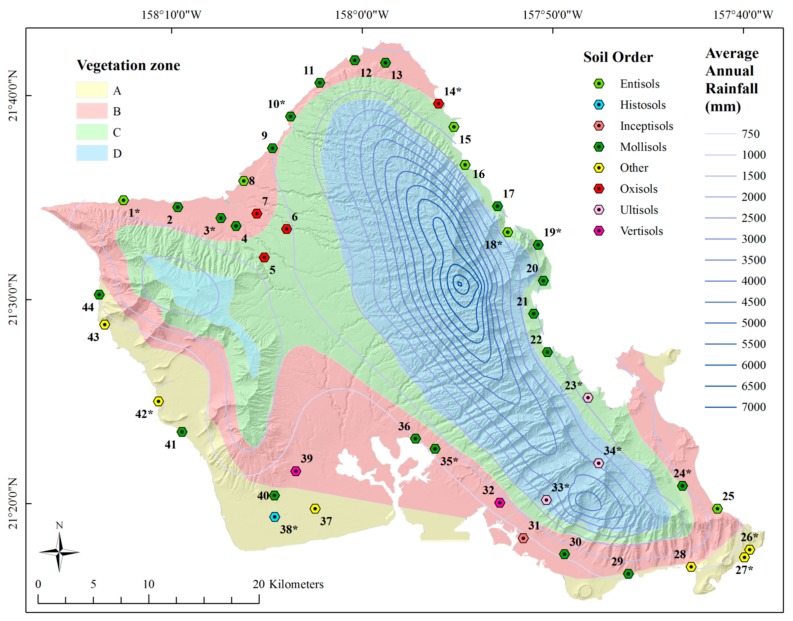
Roadside survey sites by vegetation zone (land divisions according to temperature and precipitation), average annual precipitation, and soil order (NAD 1983 4N; WGS 1984). * indicate sites with paired near and far sites.

A systematic survey of ants was conducted along major roads on the island of Oahu, Hawaii from September to November 2012 (Figure 1).

### 2.1. Study Location

Oahu is the third largest and most populous island of Hawaii, with about 80% of the state’s population [27]. Two major mountain chains span the island: the Koolau Range on the eastern coast (~50–950 m high by ~72 km long) and the Waianae Range on the western coast (~470–1200 m by ~56 km long) [27]. Oahu has two primary physiographic zones, windward and leeward, with higher precipitation on the windward side [28]. Small variation in solar radiation, buffering of the ocean, and the effect of trade winds contribute to Oahu’s mild temperatures, with an average temperature of 25 °C (21–29 °C) [28,29]. Oahu has a colder, wetter season from October to April (average temperature 24.1 ± 1.3 °C) and a warmer season from May to September (average temperature 26.9 ± 0.9 °C) [28].

Ripperton and Hosaka [30] characterized five major vegetation zones in Hawaii primarily to describe areas with similar climate, soils and vegetation types for use in agriculture [18]. Vegetation zones are highly influenced by precipitation. Since these factors also influence ant distribution, they are of interest as an explanatory variable for ant distributions.

Soil type is also of interest as an explanatory variable for ant distributions. Soil orders are classified based on several factors (e.g., pH and particle size, etc.). In Portugal, certain soils were suggested to prevent colonization of *L. humile* [31]. Ant species composition and abundance differed in serpentine and non-serpentine soils in California chaparral ecotypes [32]. Further, leaf-cutting ants were found to have a preference for Oxisols [33]. Nine soil orders occur on Oahu [34]. By acreage, Oxisols are the most common order, followed by Ultisols, Mollisols, and Vertisols [34]. Oxisols are infertile, weathered soils found at varying elevations and degrees of precipitation; Ultisols are weathered soils in wetter areas; Mollisols are nutrient-rich soils common in grasslands and coastal plains; and Vertisols are clay-rich soils in dry, lowland regions [34].

### 2.2. Mapping of Points

A random starting point between 0 to 1000 m from the beginning of major roads (Farrington Highway, Kalanianaole Highway, Kamehameha Highway, Kaukonahua Road, King Street, Pali Highway, Roosevelt Avenue) was selected. From this starting point, possible collection points were generated at 1 km intervals using “Construct Points” in ArcMap 10.1 [35]. Points were numbered and evaluated for suitability in Google Earth [36] street view. Suitable points were areas that were not on private property and safe (e.g., not on a steep cliff or dangerously close to traffic).

Starting from the random point, every fourth point was selected (4 km intervals). Points considered unsuitable were replaced with the nearest suitable point (1 to 2 km away from original selection) or discarded entirely if no replacement was accessible. Points that fell within state parks where a permit was denied (Kaena Point) were also discarded. Recent studies that have focused on roadside distributions include Gippet et al. [37].

In total, 44 sites were found suitable for collecting ants (Figure 1; Table 1); the mean temperature during collection was 25.7 ± 1.4 °C.

### 2.3. Collection

Special use application permits were obtained for collecting ants at points that fell within state parks from the State of Hawaii’s Department of Land and Natural Resources Division of State Parks. No permits were necessary for city parks.

A timed search (one person, 30 min) for ants occurred once at each suitable site (“near” sites). A 15 m tape measure was laid down starting from the edge of the pavement or edge of a barrier (e.g., stone wall or fence) in a perpendicular direction. Collection for near sites occurred within the 15 m distance. The area searched varied from 25 to 450 m^2^, depending on the layout of the site and searchable substrates; however, all searches were of the same duration (30 min). 

To address possible convenience sampling bias, additional collections (one person, 30 min) occurred beyond the 15 m distance for a subset of sites (“far” sites) and adjacent to the near sites. The area searched for far sites varied from 40 to 750 m^2^, and the width of the sites varied from 2 to 30 m. Larger areas searched were usually due to large expanses of grass or sand between searchable substrates; however, all far site searches were of the same duration. It is assumed that these subsets of paired near and far sites are similar in geography, average annual precipitation, elevation, and soil type, and generally only differ in the degree of road disturbance.

Ants were collected from plants (e.g., flowers, leaves, trunks, twigs, etc.), the ground (e.g., leaf litter, concrete, grass, etc.), objects (e.g., walls, telephone poles, trash cans, etc.), and under objects (e.g., rocks, logs, trash, etc.) [38]. Fallen branches with evidence of insect damage were opened to extract ants. Ants were collected by hand with an aspirator, forceps or scooped up within a tube, with the number of ants ranging from 1 to 20 individuals per tube, with at least one tube per substrate sampled. Specimens were stored in 95% ethyl alcohol. To ensure collections occurred during periods of peak ant activity, collection times were restricted to between 8:00 a.m. to 11:00 a.m. and 1:00 p.m. to 4:00 p.m.

### 2.4. Identification

Ants were identified to species using the Hawaii Ant Lab’s key [39], the Pacific Invasive Ant Key [40], and Bolton’s revision of *Technomyrmex* [41]. Ants were identified using a dissecting microscope. Voucher specimens are located at the corresponding author’s residence.

### 2.5. Mapping

Latitude, longitude, and altitude at each collection site were taken with a Garmin GPS 76 (mean accuracy 8.0 ± 4.9 m). Coordinates were then re-checked with Google Earth [36] for accuracy. Sites were labeled sequentially to get map codes.

Map layers were downloaded from the State of Hawaii Office of Planning GIS Data site, the University of Hawaii Geography Department, and the United States Department of Agriculture Natural Resources Conservation Service’s Geospatial Data Gateway [42,43,44,45]. Honolulu County Land Cover data were downloaded from the NOAA Coastal Services Center [46]. Average precipitation (mm), elevation contours (10 m), and soil data were used to create maps and to spatially join data to points. Layers available as raster data were first converted to points and spatially joined with ArcMap 10.1 [35]. Vegetation zones were mapped with ArcMap 10.1 [35] using Ripperton and Hosaka’s map [30] as an overlay.

### 2.6. Analysis

For each ant species occurring at a minimum of 20% (9 or more) of sites, a logistic regression was performed on the presence/absence of the ant species at each site with elevation, average annual precipitation, and soil order as explanatory variables. For the same set of species, a regression was performed on the presence/absence of the ant species at each site with vegetation zone as an explanatory variable. All analyses were done using JMP [47] unless otherwise mentioned.

The number of species in the presence and absence of dominant species [6,48] occurring at 20% of sites or more (*P. longicornis*, *P. megacephala*, and *Technomyrmex difficilis* Forel) was subjected to a *t*-test to compare each species’ impact on the ant assemblages.

Finally, species occurrences for paired near and far sites were tabulated. A paired *t*-test was performed on species richness for the 15 pairs of near and far sites. A multi-response permutation procedure (MRPP) analyzing the presence and absence of ant species at near and far sites using natural weighting (C_1_ = n_1_/Σn_1_) was done in PC-ORD 5 [49] to compare species compositions. The Jaccard coefficient, *J*, [50] measures similarity between two sets of presence/absence data, here comparing near and far samples:

*J = S/[S + N + F]*

where *S* = shared near and far species, *N* = unique near species, and *F* = unique far species. The Jaccard distance,

*D*, (*D =* 1 − *J*)

was calculated for each pair of near and far samples to compare differences in species composition. The overall Jaccard distance for all near and far sites was also calculated. Real’s [51] table of critical values for Jaccard’s index of similarity was used to check for significantly different than random values for Jaccard distances [52].

## 3. Results

### 3.1. Road Survey Results

Twenty-four species of ants from five subfamilies (Dolichoderinae, Formicinae, Myrmicinae, Ponerinae, and Pseudomyrmecinae) were found from the 44 sites (Table 2). *Solenopsis invicta* Buren (not reported in Hawaii), *W. auropunctata*, and *L. humile*, were not observed at any sites in this study. The distributions of white-footed ants (*T. albipes*, *T. difficilis*, *T. pallipes*, and *Technomyrmex vitiensis* Mann) show *T. difficilis* is the most widespread of the suite (Table 2). None of the ants found in the present survey are new records.

The relationship between ant presence and elevation, precipitation, and soil order is given in Table 3. Ochetellus glaber was found in areas with less precipitation (*p* = 0.01) around Oahu. Paratrechina longicornis was found more often in lower elevations (*p* = 0.01) and in areas with the Mollisol soil order (*p* = 0.01). Tetramorium simillimum (Smith, F.) was found in lower elevations (*p* = 0.02) and in areas with the Mollisol soil order. *Pheidole megacephala* was found more often in vegetation zone C (*p* = 0.01), while *O. glaber*, *T. difficilis*, *P. longicornis*, *Plagiolepis alluaudi* Emery, and *T. simmillimum* did not significantly differ by vegetation zone.

The presence of *P. megacephala* is associated with fewer species of ants per site (Table 4). *Paratrechina longicornis* and *T. difficilis* were both associated with significantly higher mean numbers of ant species per site (Table 4).

### 3.2. Convenience Sampling Results

A total of 21 species of ants from five subfamilies were collected from 15 pairs of near and far sites (Table 5).

Species richness did not significantly differ by site type (near vs. far). The MRPP indicated that, overall, species composition did not significantly differ between near and far sites (*t* = 1.05, *p* = 0.89). Although some sites showed differences in the species compositions of ants in near and far sites, using Real’s [51] table of significant values of Jaccard’s index of similarity, most did not differ significantly. Lastly, the overall Jaccard distance (33.33%) for all near and far sites combined was not significant.

## 4. Discussion

The distributions of 24 species of ants were mapped on Oahu roadsides, contributing to continued documentation of ants in Hawaii. *Pheidole megacephala* was the dominant ant of roadsides on Oahu. Two recent invaders, *O. glaber* and *T. difficilis*, were among the most frequently encountered ants.

*Pheidole megacephala* was found more often on the windward side of Oahu, which is consistent with its correlation with vegetation zone C. *Pheidole megacephala* was also associated with fewer species of ants where it occurred, possibly because of its aggressive behavior.

*Technomyrmex difficilis* is rapidly expanding its range outside its native Madagascar [48]. *Technomyrmex difficilis* is considered by Wetterer [48] to be a dominant arboreal pest in the West Indies and Florida. However, the mean number of ants in the presence of *T. difficilis* is higher than in its absence. Possible reasons include its relatively recent establishment and possible lack of overlap of resources with other species of ants.

*Ochetellus glaber*, the glaber ant, was first noted in 1977 by Beardsley [53] at Hickam Air Force Base on Oahu. This ant appears to be widely distributed around the island with a preference for areas with less precipitation. *Ochetellus glaber* occurred with *P. megacephala* at ten sites and *S. geminata* at three sites, suggesting it can persist even with strongly competitive species. 

Two ants were found more often in areas with Mollisol soil (*P. longicornis* and *T. simillimum*). However, nearly half of all sites had Mollisol soil, which may reduce the explanatory power of soil type in this study.

The tropical fire ant, *S. geminata*, was found at seven sites. The red imported fire ant, *S. invicta*, is known to displace *S. geminata* where it occurs [54], and the sites where *S. geminata* now occurs should be monitored closely for signs of possible introduction and establishment of *S. invicta.*

Though *M. pharaonis* was first recorded in 1913 [16], both Huddleston and Fluker [18] and the present study failed to collect this species, possibly due to the ecology of this ant and the collection methodology employed. The Hawaii Department of Health Vector Control Branch received more complaints about *M. pharaonis* than any other ant species [7]. However, BYU [22] collected this species via hand-collecting.

It was expected that less disturbed areas (far sites) would have more species than disturbed areas (near sites). However, the paired *t*-test showed no significant difference between species richness of near and far sites. This may be due to the small sample size (n = 15) of paired near and far sites that would require a large effect size for statistical significance. The species compositions of near and far sites were not significantly different, suggesting species assemblages near and further from roads are similar according to this sampling protocol.

Several limitations are inherent in this study. All sites were close to roads, and thus human disturbance and urbanization [12]. Also most sites were near coastlines, and at lower elevations, which limits the representation of ants in differing ecological communities (e.g., montane, cool dry forest, alpine scrub, etc. [12]). The ants collected do not represent the full assemblage of ants present on Oahu, as all sites were from roadsides, and cryptic and nocturnal species may not have been collected. The recent introduction of certain ant species, which have not yet attained their total potential distributions, may skew analyses relating to correlation with other species and with environmental factors.

However, an update to the hand-collection surveys is useful, as nearly twenty species have been added to Hawaii’s ant fauna since Huddleston and Fluker’s [18] island-wide hand-collection survey. The current study also shows a possible decrease in ants that were previously frequently found (e.g., *A. gracilipes*, *L. humile*, *T. bicarinatum*, etc.). The red imported fire ant, *S. invicta*, little fire ant, *W. auropunctata*, and the Argentine ant, *L. humile*, were not found in the present survey, and no new species of ants were encountered; however, the little fire ant has recently been found in Waimanalo and Mililani Mauka on Oahu [55]. 

These data provide a useful comparison for future studies. Surveys sampling a wider range of elevations and soil types and away from roadsides are needed though to solidify possible associations with ant distributions. 

## Figures and Tables

**Table 1:** Site summary of abiotic factors by vegetation zone (land divisions according to temperature and precipitation) [30] for a timed hand-collection of ants in 2012.

Vegetation Zone	No. of Sites	Elevation (m)		Average Annual Precipitation (mm)	
		Mean ± SD	Range	Mean ± SD	Range
A	11	8 ± 10	1 to 35	658 ± 94	541 to 784
B	18	12 ± 21	1 to 82	876 ± 156	615 to 1194
C	13	39 ± 84	1 to 234	1370 ± 266	934 to 1767
D	2	237 ± 178	111 to 362	2198 ± 362	1942 to 2454
Total	44		1 to 362		541 to 2454

**Table 2:**
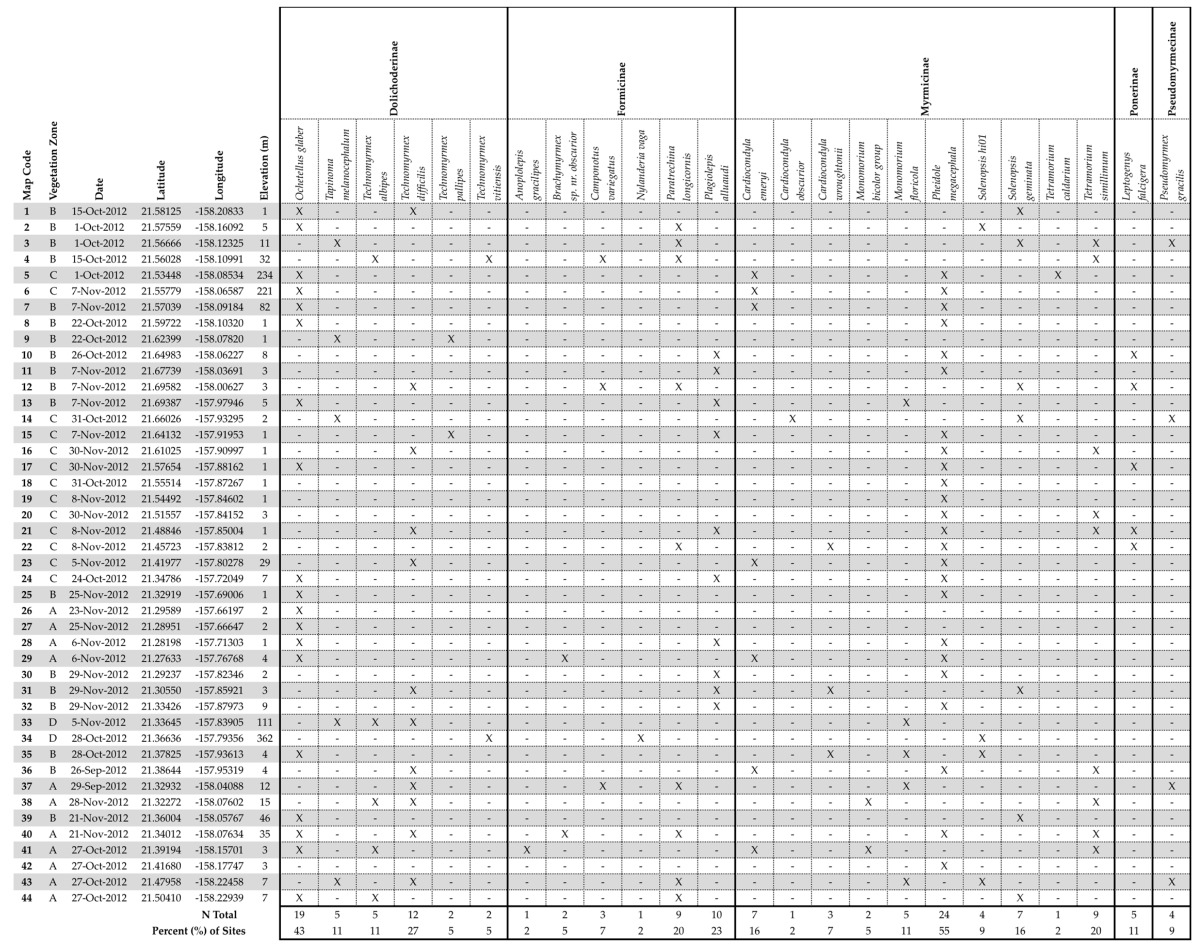
Ant species collected from 44 sites on Oahu, Hawaii from a timed hand-collection survey in 2012. Vegetation zone (land division according to temperature and precipitation) is from Ripperton and Hosaka [30].

**Table 3:** Relationship between ant presence and elevation, precipitation, and soil conditions on Oahu.

	*p*-Value from Logistic Regression			
Species (No. of Sites)	Whole Model	Elevation	Precipitation	Soil
**Dolichoderinae**				
Ochetellus glaber (19)	0.03 *	0.22	0.01 *	0.30
Technomyrmex difficilis (12)	0.10	-	-	-
**Formicidae**				
Paratrechina longicornis (9)	0.01 *	0.01 *	0.62	0.01 *
Plagiolepis alluaudi (10)	0.19	-	-	-
**Myrmicinae**				
Pheidole megacephala (24)	0.34	-	-	-
Tetramorium simillimum (9)	0.04 *	0.02 *	0.46	0.02 *

* Indicate significant *p*-values.

**Table 4:** Mean number of ant species in the presence and absence of three ant species on Oahu from a timed hand-collection survey in 2012.

Species	N sites Species Is Present	N Sites Species Is Absent	Mean Species Richness (±SD) Where Species Is Present	Mean Species Richness (±SD) Where Species Is Absent	*p*-Value
*Paratrechina longicornis*	9	35	4.78 ± 0.97 a	2.86 ± 1.19 b	0.0001
*Pheidole megacephala*	24	20	2.88 ± 1.23 a	3.70 ± 1.46 b	0.0259
*Technomyrmex difficilis*	12	32	4.33 ± 1.07 a	2.84 ± 1.27 b	0.0004

Means within the same row followed by different letters indicate significant differences.

**Table 5:** Species occurrences of ants in paired near and far road sites from a hand-collection survey in 2012 and Jaccard distances by site number.

Site No.	Unique Near Species	Species at Both Near and Far Sites	Unique Far Species	Jaccard Distance
**1**	*Solenopsis geminata* *Technomyrmex difficilis*	*Ochetellus glaber*	*Pheidole megacephala* *Plagiolepis alluaudi*	75.00%
**3**	*Paratrechina longicornis* *Pseudomyrmex gracilis* *Tetramorium simillimum*	*Solenopsis geminata* *Tapinoma melanocephalum*	*Cardiocondyla emeryi* *Ochetellus glaber*	71.43%
**10**		*Leptogenys falcigera* *Pheidole megacephala* *Plagiolepis alluaudi*	*Cardiocondyla obscurior* *Ochetellus glaber* *Paratrechina longicornis*	40.00%
**14**	*Pseudomyrmex gracilis*	*Cardiocondyla obscurior* *Solenopsis geminata* *Tapinoma melanocephalum*	*Monomorium floricola* *Technomyrmex albipes* *Tetramorium simillimum*	57.14%
**18**		*Pheidole megacephala*		0.00%
**19**		*Pheidole megacephala*		0.00%
**23**	*Cardiocondyla emeryi*	*Pheidole megacephala* *Technomyrmex difficilis*	*Plagiolepis alluaudi*	50.00%
**24**	*Plagiolepis alluaudi*	*Ochetellus glaber* *Pheidole megacephala*	*Cardiocondyla emeryi*	50.00%
**26**		*Ochetellus glaber*		0.00%
**27**		*Ochetellus glaber*	*Pheidole megacephala* *Plagiolepis alluaudi*	66.67%
**33**	*Tapinoma melanocephalum* *Technomyrmex difficilis*	*Monomorium floricola* *Technomyrmex albipes*	*Cardiocondyla obscurior* *Leptogenys falcigera* *Pheidole megacephala* *Technomyrmex vitiensis*	75.00%
**34**	*Nylanderia vaga* *Solenopsis hi01* *Technomyrmex vitiensis*			100.00% *
**35**	*Cardiocondyla wroughtonii* *Monomorium floricola* *Ochetellus glaber* *Solenopsis hi01*		*Cardiocondyla emeryi* *Cardiocondyla obscurior* *Solenopsis geminata* *Technomyrmex vitiensis*	100.00% *
**38**	*Monomorium bicolor group* *Technomyrmex difficilis* *Tetramorium simillimum*	*Technomyrmex albipes*	*Camponotus variegatus*	80.00%
**42**		*Pheidole megacephala*	*Ochetellus glaber* *Monomorium destructor* *Technomyrmex difficilis*	75.00%

Jaccard distances with * indicate *p* < 0.05.

## Supplementary Materials

The following are available online at http://www.mdpi.com/2075-4450/9/1/21/s1, Figure S1: Ant species collected from a roadside survey on Oahu, Hawaii in 2012.

## References

[1] Hölldobler B., Wilson E.O. (1990). The Ants.

[2] Holway D.A., Lach L., Suarez A.V., Tsutsui N.D., Case T.J. (2002). The causes and consequences of ant invasions. Ann. Rev. Ecol. Syst..

[3] Hill J.G., Summerville K.S., Brown R.L. (2008). Habitat associations of ant species (Hymenoptera: Formicidae) in a Heterogeneous Mississippi landscape. Environ. Entomol..

[4] Chong K.-F., Lee C.-Y. (2010). Inter- and intraspecific aggression in the invasive Longlegged Ant (Hymenoptera: Formicidae). J. Econ. Entomol..

[5] Gutrich J.J., VanGelder E., Loope L. (2007). Potential economic impact of introduction and spread of the red imported fire ant, Solenopsis Invicta, in Hawaii. Environ. Sci. Policy.

[6] Krushelnycky P.D., Loope L.L., Reimer N.J. (2005). The ecology, policy, and management of ants in Hawaii. Proc. Hawaii. Entomol. Soc..

[7] Leong M.H.K., Grace J.K. (2008). Occurrence and distribution of ants (Hymenoptera: Formicidae), cockroaches (Blattodea), centipedes (Chilopoda) and wasps (Hymenoptera: Vespidae) of public health importance on the island of Oahu. Proc. Hawaii. Entomol. Soc..

[8] Pantoja L.D.M., Filho R.E.M., Brito E.H.S., Aragão T.B., Brilhante R.S.N., Cordeiro R.A., Rocha M.F.G., Monteiro A.J., Quinet Y.P., Sidrim J.J.C. (2009). Ants (Hymenoptera: Formicidae) as carriers of fungi in hospital environments: An emphasis on the Genera *Tapinoma* and *Pheidole*. J. Med. Entomol..

[9] Rust M.K., Su N.-Y. (2012). Managing social insects of urban importance. Ann. Rev. Entomol..

[10] Howarth F.G., Sohmer S.H., Duckworth W.D. (1988). Hawaiian natural history and conservation efforts: What’s left Is worth saving. BioScience.

[11] Wilson E.O., Taylor R.W. (1967). The Ants of Polynesia (Hymenoptera, Formicidae)—Pacific Insects Monograph.

[12] Reimer N.J., Williams D.F. (1994). Distribution and impact of alien ants in vulnerable Hawaiian ecosystems. Exotic Ants: Biology, Impact, and Control of Introduced Species.

[13] AntWeb: Ants of Hawaii. https://www.antweb.org/adm1.do?name=Hawaii&country=United+States.

[14] Forel A., Sharp D. (1899). Heterogyna (Formicidae). Fauna Hawaiiensis.

[15] Wheeler W.M. (1934). Revised list of Hawaiian ants. Occasional Papers of the Bernice Pauahi Bishop Museum of Polynesian Ethnology and Natural History.

[16] Gulick L. (1913). Synoptic list of ants reported from the Hawaiian Islands. Proc. Hawaii. Entomol. Soc..

[17] Phillips J.S. (1934). The Biology and Distribution of Ants in Hawaiian Pineapple Fields.

[18] Huddleston E.W., Fluker S.S. (1968). Distribution of ant species of Hawaii. Proc. Hawaii. Entomol. Soc..

[19] Reimer N., Beardsley J.W., Jahn G. (1990). Pest ants in the Hawaiian islands. Applied Myrmecology—A World Perspective.

[20] Plentovich S. (2010). Appendix 7-2: Final Report: Survey of Invasive Ant Species within Makua and Oahu Implementation Plan Management Units, Oahu, Hawaii 2004–2009.

[21] Heu R.A., Suh T.H. (2006). Red Imported Fire ant Semiannual Report FY 2005.

[22] Martin C.F. (2012). A Survey of Invasive Exotic Ants Found on Hawaiian Islands: Spatial Distributions and Patterns of Association. Master’s Thesis.

[23] Morrison M.L. (2012). The Habitat Sampling and Analysis Paradigm Has Limited Value in Animal Conservation: A Prequel. J. Wildl. Manag..

[24] Johnston F.M., Johnston S.W. (2004). Impacts of Road Disturbance on Soil Properties and on Exotic Plant Occurrence in Subalpine Areas of the Australian Alps. Arct. Antarct. Alp. Res..

[25] Niemelä J., Kotze D.J., Venn S., Penev L., Stoyanov I., Spence J., Hartley D., de Oca E.M. (2002). Carabid Beetle Assemblages (Coleoptera, Carabidae) across Urban-Rural Gradients: An International Comparison. Landsc. Ecol..

[26] Vonshak M., Gordon D.M. (2015). Intermediate disturbance promotes invasive ant abundance. Biol. Conserv..

[27] Hartley T.M., Chen Y.L. (2010). Characteristics of summer trade wind rainfall over Oahu. Weather Forecast..

[28] Brasher A.M.D., Wolff R.H., Luton C.D. (2003). Associations among Land Use, Habitat Characteristics, and Invertebrate Community Structure in Nine Streams on the Island of Oahu, Hawaii, 1999–2001.

[29] U.S. Climate Data. http://www.usclimatedata.com/climate/hawaii/united-states/3181.

[30] Ripperton J.C., Hosaka E.Y. (1942). Vegetation Zones of Hawaii.

[31] Way M.J., Cammell M.E., Paiva M.R., Collingwood C.A. (1997). Distribution and dynamics of the argentine ant Linepithema (Iridomyrmex) Humile (Mayr) in relation to vegetation, soil conditions, topography and native competitor ants in Portugal. Insectes Sociaux.

[32] Fisher B.L. (1997). A comparison of ant assemblages (Hymenoptera, Formicidae) on serpentine and non-serpentine soils in Northern California. Insectes Sociaux.

[33] Schoereder J.H., DaSilva W.L. (2008). Leaf-cutting ants (Hymenoptera: Formicidae) and soil classes: Preference, survival and nest density. Sociobiology.

[34] Deenik J., McClellan A.T. (2007). Soils of Hawaii.

[35] ESRI (2013). ArcGIS Desktop: Release 1.

[36] Google, Inc. (2013). Google Earth: Version 7.0.3.8542.

[37] Gippet J.M.W., Mondy N., Diallo-Dudek J., Bellec A., Dumet A., Mistler L., Kaufmann B. (2017). I’m Not Like Everybody Else: Urbanization Factors Shaping Spatial Distribution of Native and Invasive Ants Are Species-Specific. Urban Ecosyst..

[38] Nelson M.L. (1993). Distribution of the Ants (Formicidae) on Kauai, Hawaii. Master’s Thesis.

[39] Vanderwoude C. Key to the Sub Families of Ants in Hawaii. http://www.littlefireants.com/hawaiiantkey0508_new.pdf.

[40] Sarnat E.M. PIAkey: Identifiction Guide to Ants of the Pacific Islands, Edition 2.0, Lucid v. 3.4. http://idtools.org/id/ants/pia/.

[41] Bolton B. (2007). Taxonomy of the dolichoderine ant genus Technomyrmex Mayr (Hymenoptera: Formicidae) based on the worker caste. Contrib. Am. Entomol. Inst..

[42] Coastal Geology Group Oahu. University of Hawaii at Manoa School of Ocean and Earth Science and Technology. http://www.soest.hawaii.edu/coasts/publications/hawaiiCoastline/oahu.html.

[43] Giambelluca T.W., Chen Q., Frazier A.G., Price J.P., Chen Y.L., Chu P.S., Eischeid J.K., Delparte D.M. (2013). Online rainfall atlas of Hawaii. Bull. Am. Meteorol. Soc..

[44] State of Hawaii Download GIS Data. Office of Planning. http://planning.hawaii.gov/gis/download-gis-data/.

[45] USDA Soil Survey Geographic (SSURGO) Database for Island of Oahu, Hawaii. https://www.nrcs.usda.gov/wps/portal/nrcs/surveylist/soils/survey/state/?stateId=HI.

[46] NOAA NOAA Coastal Services Center Land Cover Analysis: Honolulu County Land Cover. https://coast.noaa.gov/digitalcoast/tools/lca.

[47] SAS Institute (2013). JMP Version 11.1.1.

[48] Wetterer J.K. (2013). Worldwide spread of the difficult white-footed ant, *Technomyrmex difficilis* (Hymenoptera: Formicidae). Myrmecol. News.

[49] McCune B., Mefford M.J. (2016). PC-ORD: Multivariate Analysis of Ecological Data.

[50] Jaccard P. (1912). The distribution of flora in the alpine zone. New Phytol..

[51] Real R. (1999). Tables of significant values of Jaccard’s index of similarity. Misc. Zool. (Barc.).

[52] Urbani C.B. (1980). A Statistical Table for the Degree of Coexistence between 2 Species. Oecologia (Berl.).

[53] Beardsley J.W. (1980). *Iridomyrmex glaber* (Mayr). Proc. Hawaii. Entomol. Soc..

[54] Wetterer J.K. (2011). Worldwide spread of the tropical fire ant, *Solenopsis geminata* (Hymenoptera: Formicidae). Myrmecol. News.

[55] State of Hawaii Department of Agriculture Little Fire Ants Found in Mililani Neighborhood. http://hdoa.hawaii.gov/blog/main/lfamililani/.

