# Supplementary figures and images for: Roadside Survey of Ants on Oahu, Hawaii
Source: Insects. 2018 Feb 11;9(1):21. doi: 10.3390/insects9010021 (PMC5872286; doi:10.3390/insects9010021)

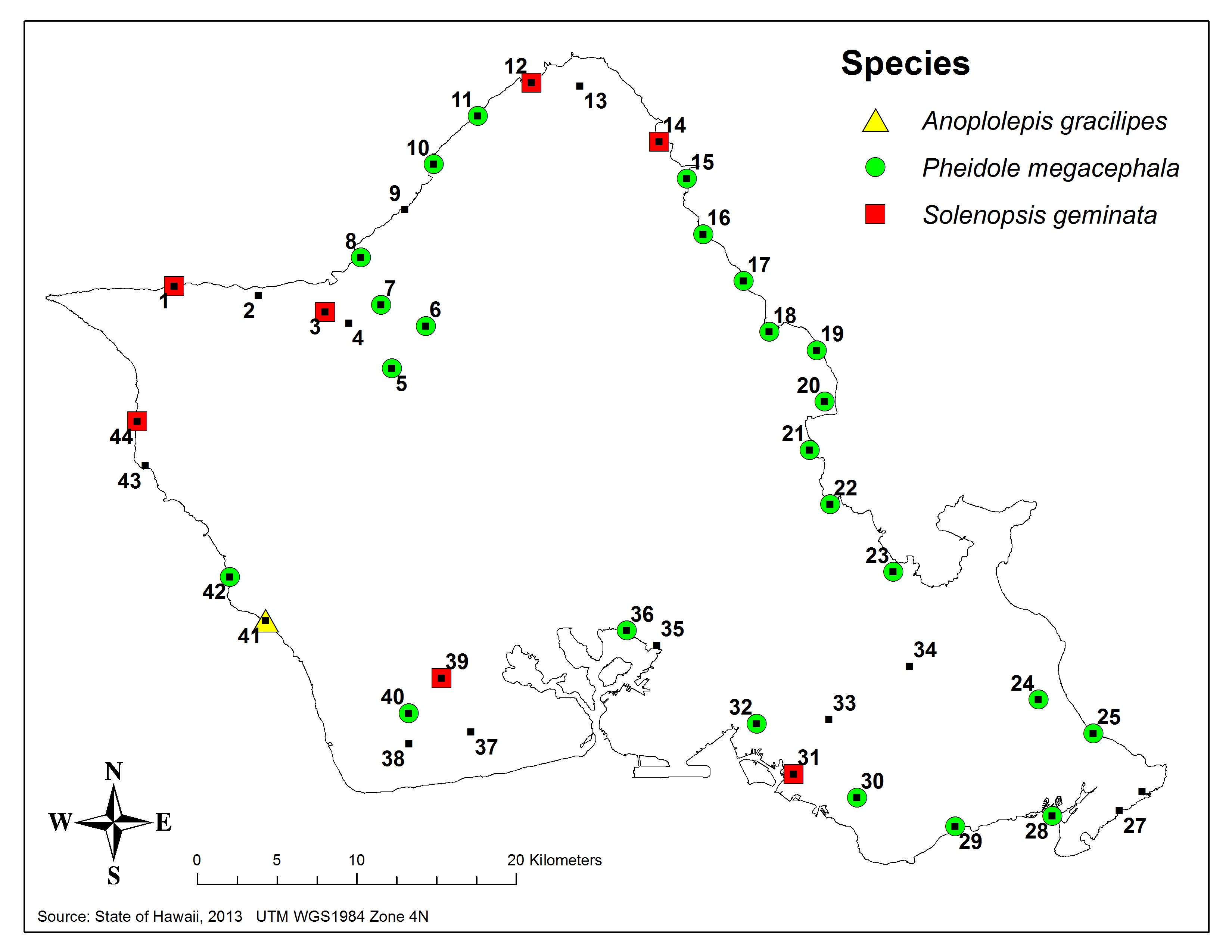

Supplement: Supplementary file 1 [file insects-09-00021-s001.zip › indmaps/anopheisolen.jpg]

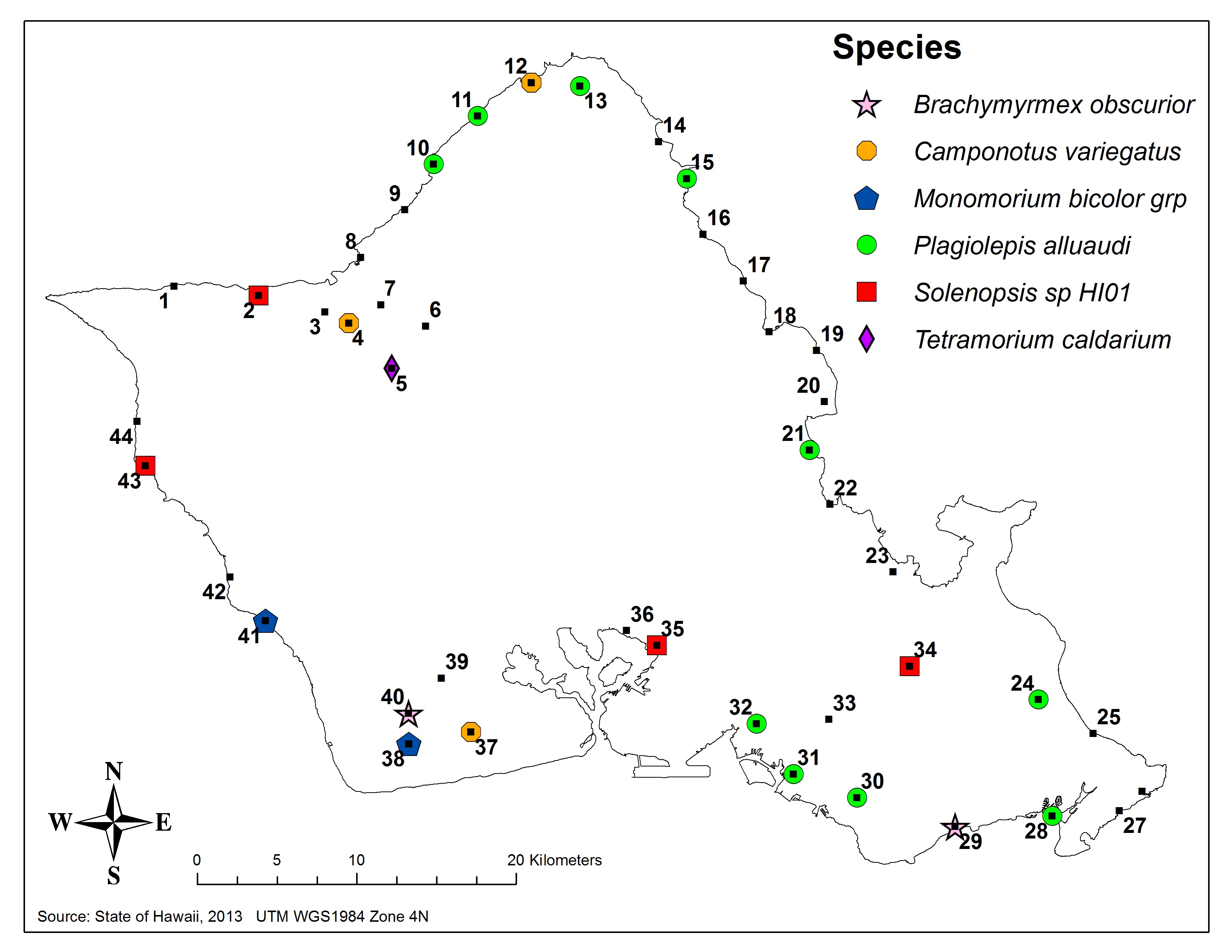

Supplement: Supplementary file 1 [file insects-09-00021-s001.zip › indmaps/brachy.jpg]

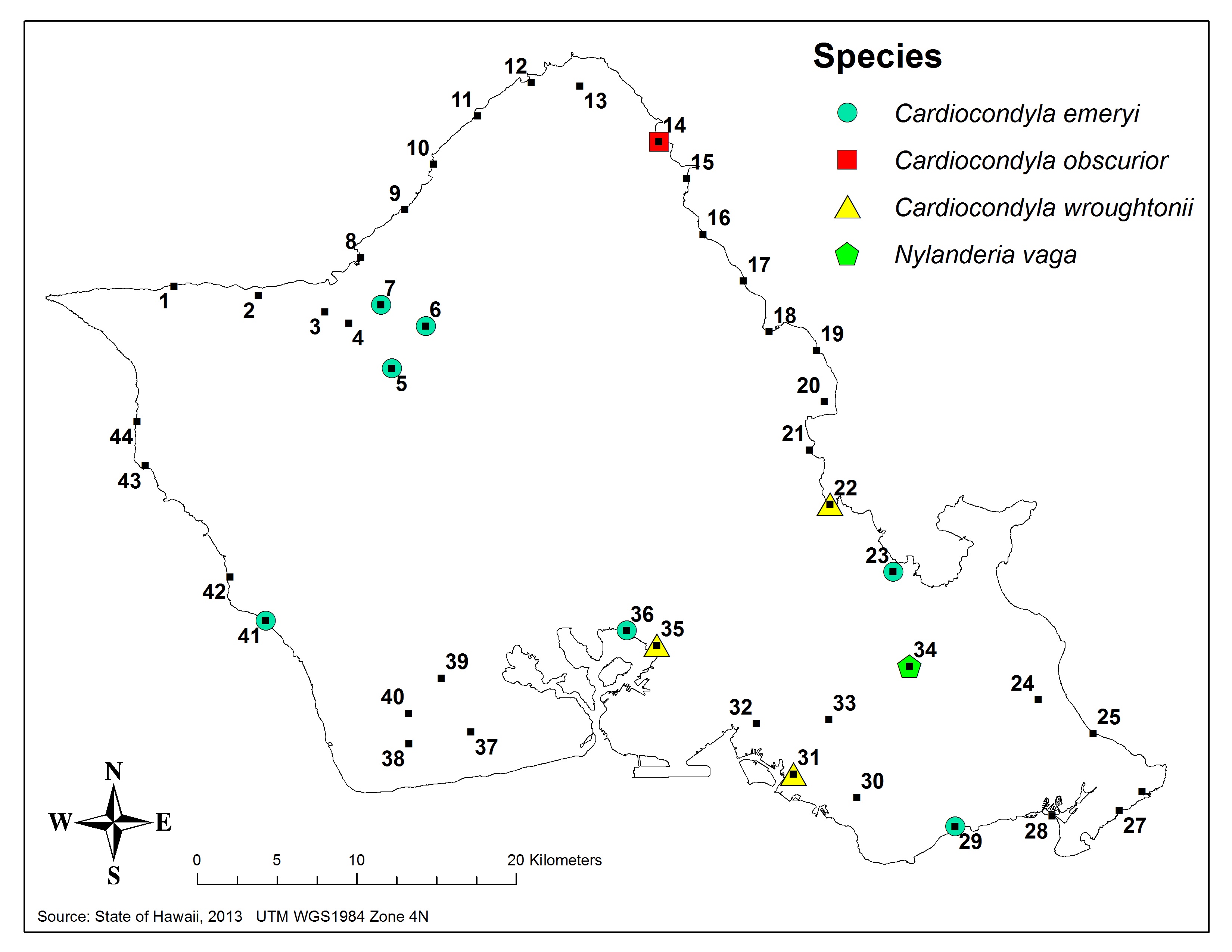

Supplement: Supplementary file 1 [file insects-09-00021-s001.zip › indmaps/cardio.jpg]

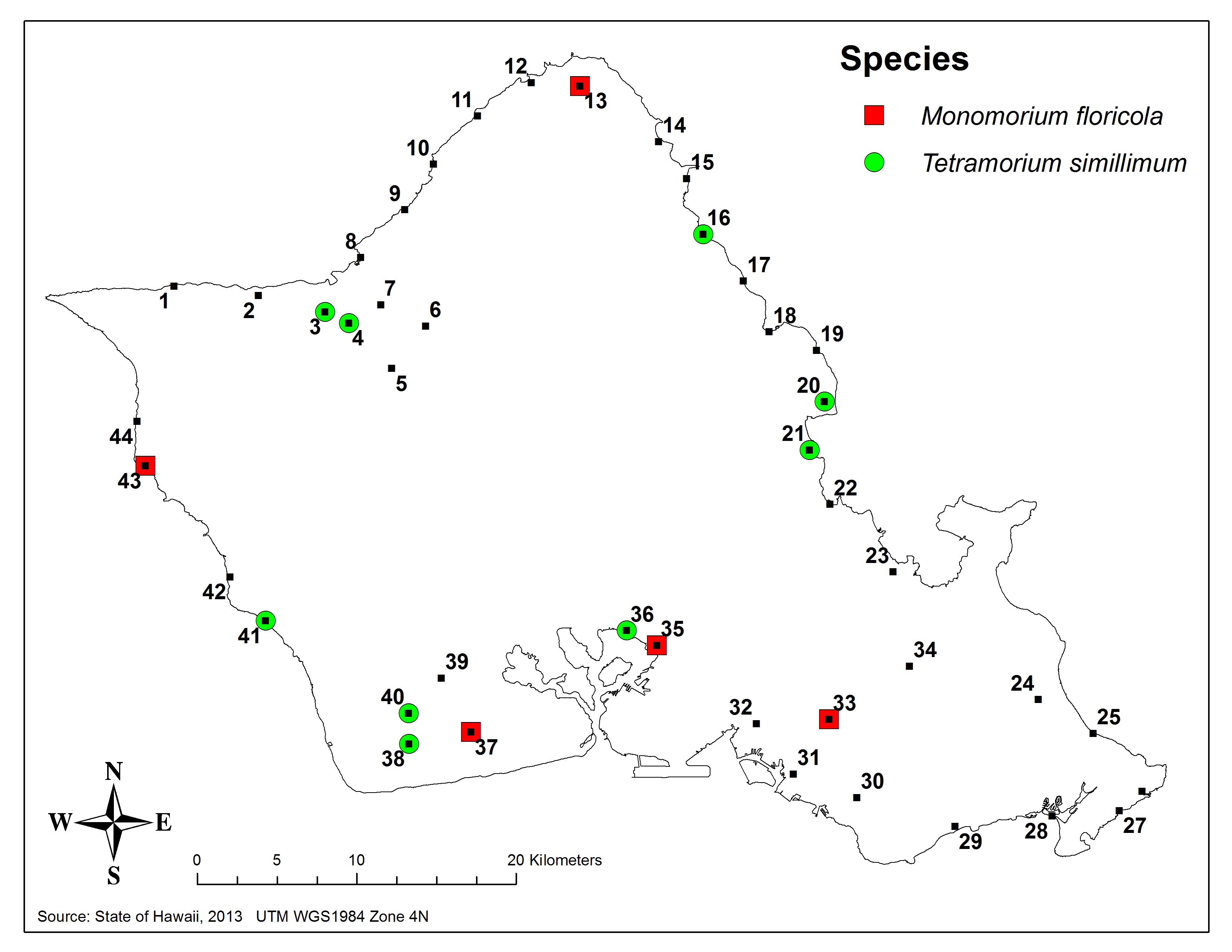

Supplement: Supplementary file 1 [file insects-09-00021-s001.zip › indmaps/florcil.jpg]

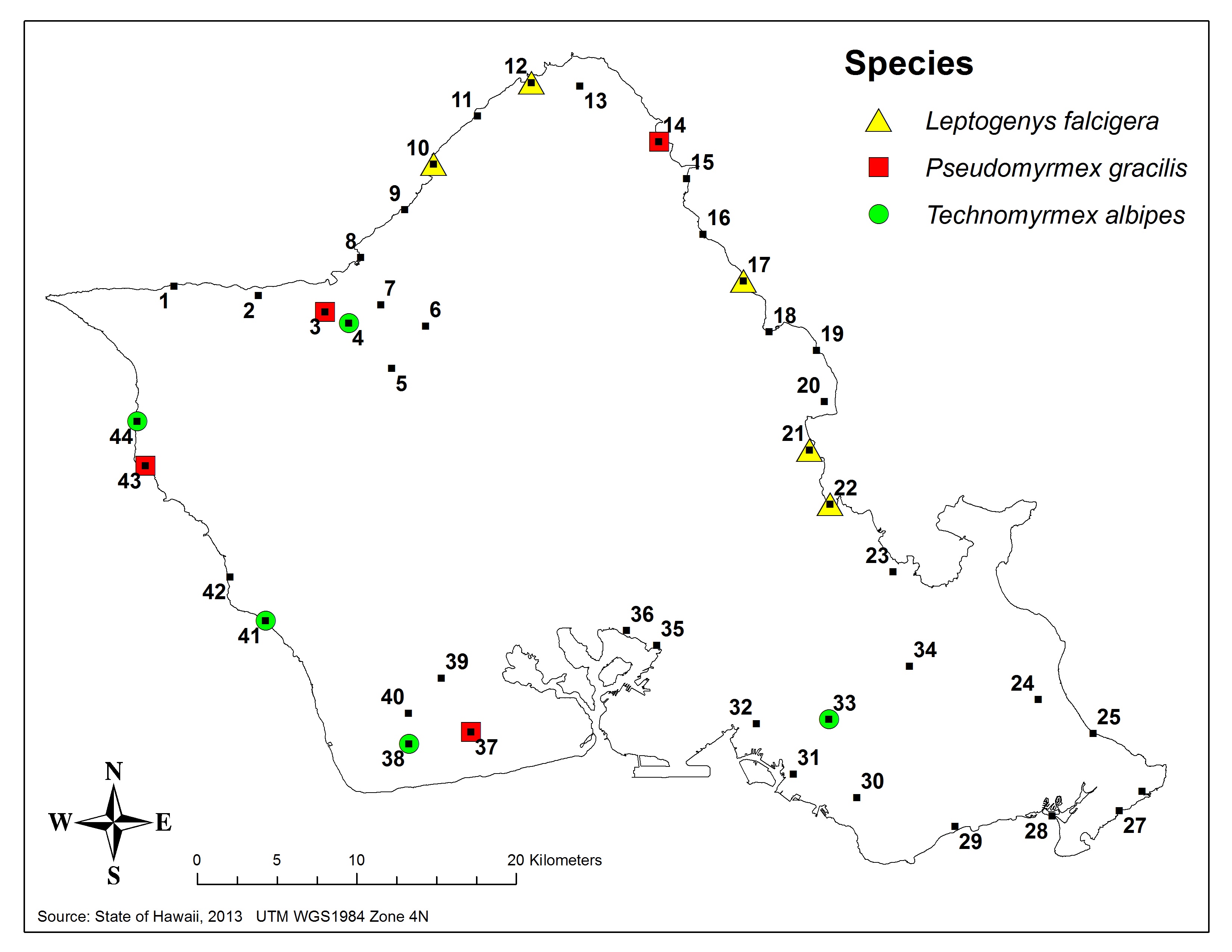

Supplement: Supplementary file 1 [file insects-09-00021-s001.zip › indmaps/lepto.jpg]

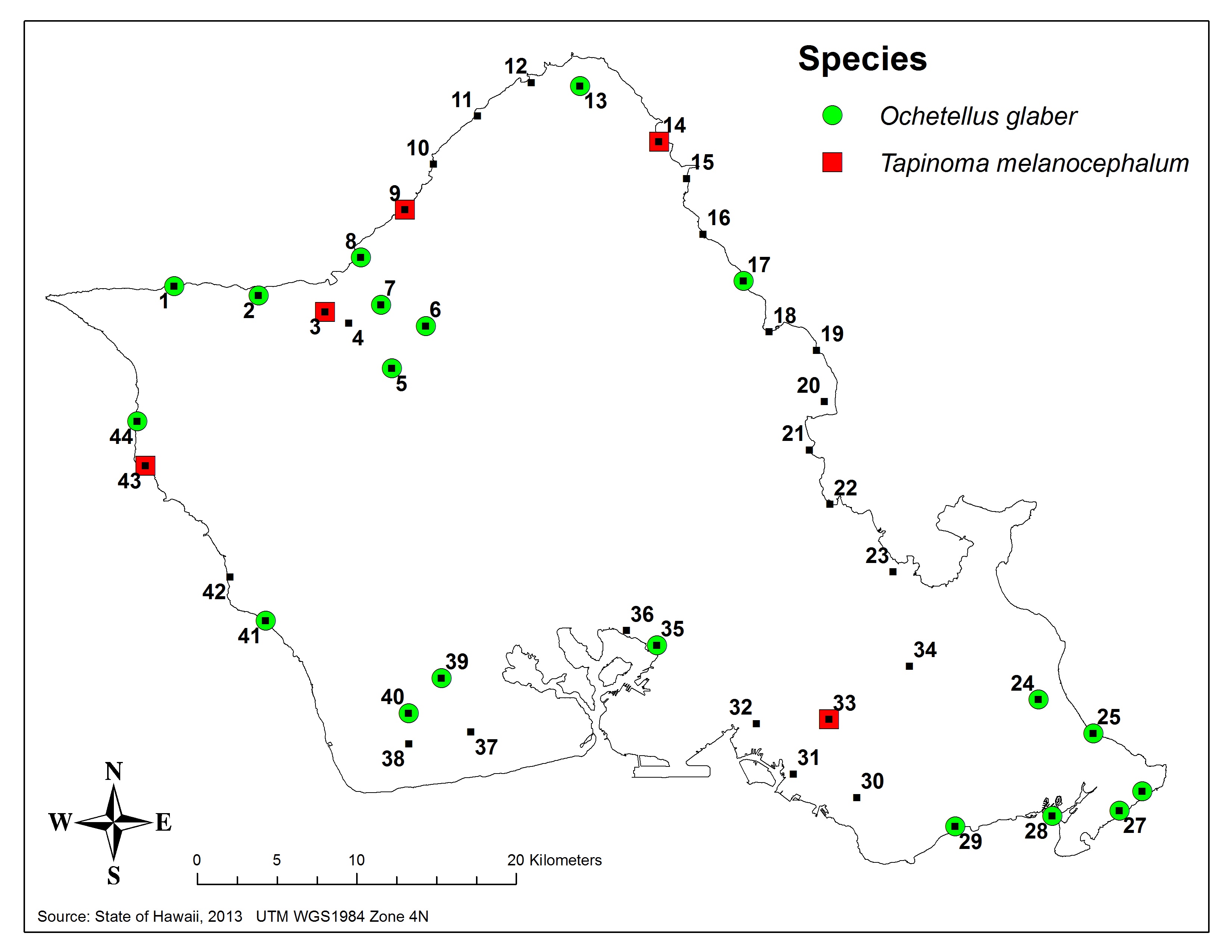

Supplement: Supplementary file 1 [file insects-09-00021-s001.zip › indmaps/ochet.jpg]

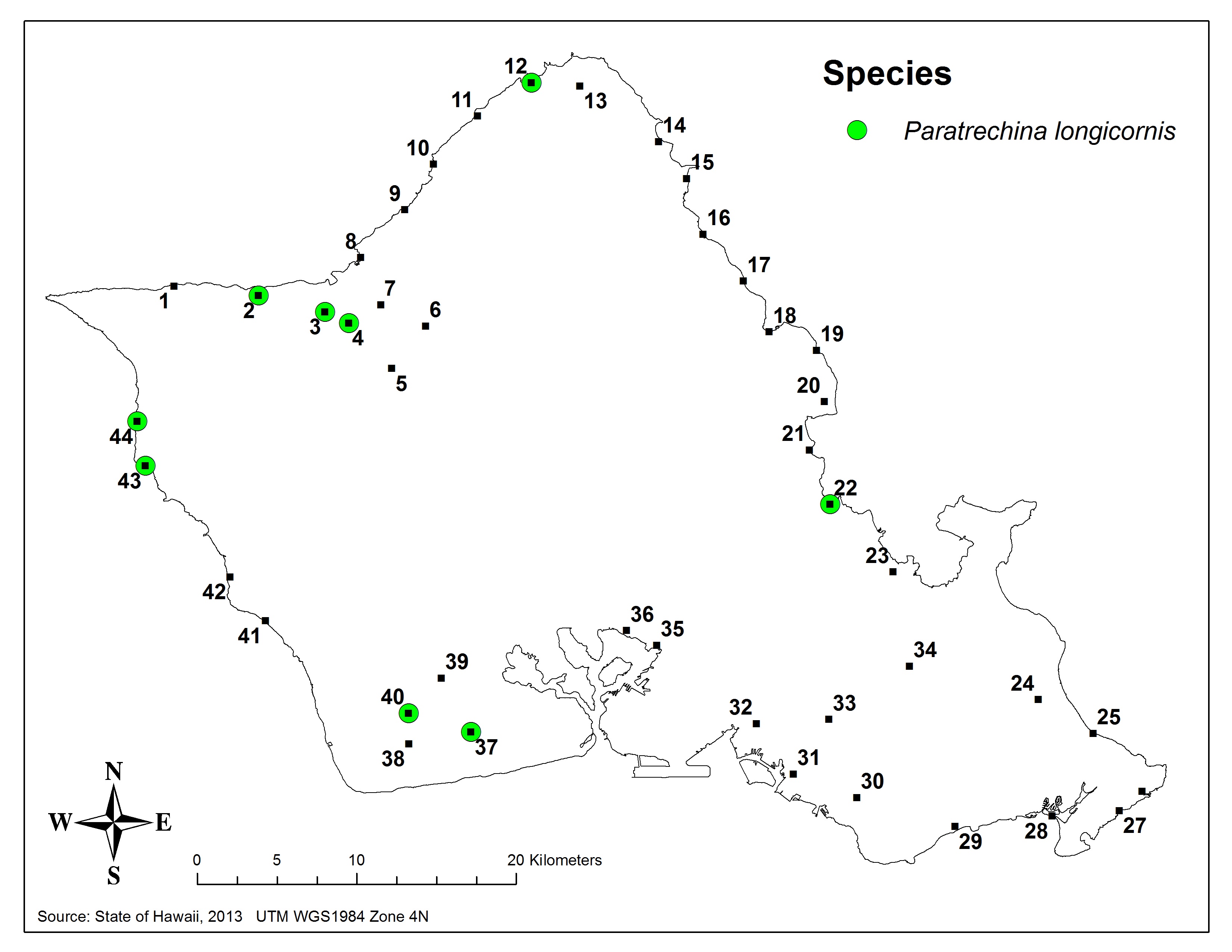

Supplement: Supplementary file 1 [file insects-09-00021-s001.zip › indmaps/parat.jpg]

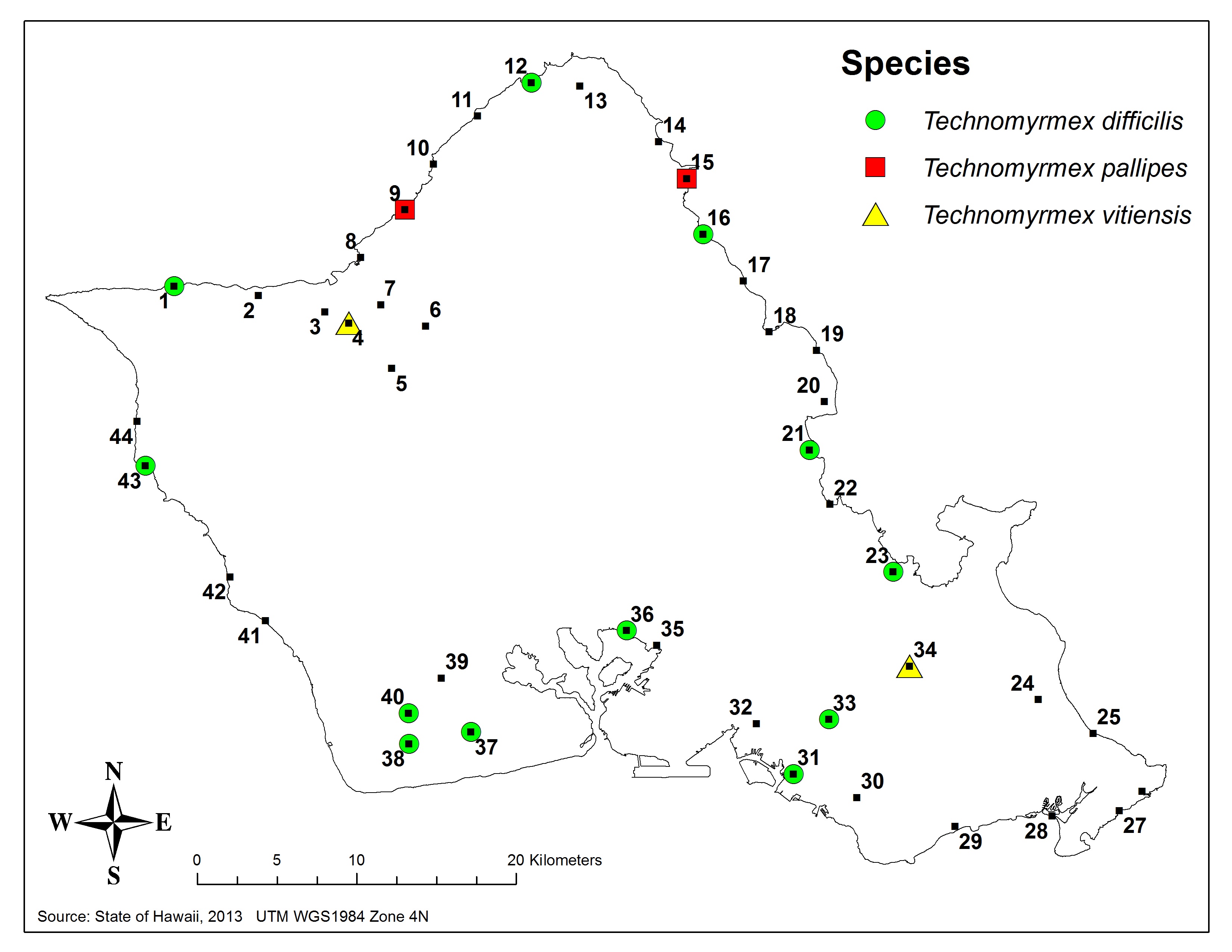

Supplement: Supplementary file 1 [file insects-09-00021-s001.zip › indmaps/techno.jpg]
